# Dysregulation of chemokine receptor expression and function in leukocytes from ALS patients

**DOI:** 10.1186/s12974-018-1135-3

**Published:** 2018-03-28

**Authors:** Caroline Perner, Florian Perner, Beatrice Stubendorff, Martin Förster, Otto W. Witte, Florian H. Heidel, Tino Prell, Julian Grosskreutz

**Affiliations:** 1 Hans Berger Department of Neurology, Jena University Hospital, Am Klinikum 1, 07747 Jena, Germany; 2 Internal Medicine II, Hematology and Medical Oncology, Jena University Hospital, Am Klinikum 1, 07747 Jena, Germany; 3 Internal Medicine I, Experimental Pneumology, Jena University Hospital, |Am Klinikum 1, 07747 Jena, Germany; 4 Leibniz-Institute on Aging – Fritz Lipmann Institute, Jena, Germany

**Keywords:** ALS, Chemokines, CXCR3, IP10, T cells

## Abstract

Amyotrophic lateral sclerosis (ALS) is rapidly progressive adult-onset motor neuron disease characterized by the neurodegeneration of both upper and lower motor neurons in the cortex and the spinal cord; the majority of patients succumb to respiratory failure. Although the etiology is not yet fully understood, there is compelling evidence that ALS is a multi-systemic disorder, with peripheral inflammation critically contributing to the disease process. However, the full extent and nature of this immunological dysregulation remains to be established, particularly within circulating blood cells. Therefore, the aim of the present study was to identify dysregulated inflammatory molecules in peripheral blood cells of ALS patients and analyze for functional consequences of the observed findings. To this end, we employed flow cytometry-based screening to quantify the surface expression of major chemokine receptors and integrins. A significantly increased expression of CXCR3, CXCR4, CCL2, and CCL5 was observed on T cells in ALS patients compared to healthy controls. Intriguingly, the expression was even more pronounced in patients with a slow progressive phenotype. To further investigate the functional consequences of this altered surface expression, we used a modified Boyden chamber assay to measure chemotaxis in ALS patient-derived lymphocytes. Interestingly, chemoattraction with the CXCR3-Ligand IP10 led to upregulated migratory behavior of ALS lymphocytes compared to healthy controls. Taken together, our data provides evidence for a functional dysregulation of IP10-directed chemotaxis in peripheral blood cells in ALS patients. However, whether the chemokine itself or its receptor CXCR3, or both, could serve as potential therapeutic targets in ALS requires further investigations.

**Electronic supplementary material:**

The online version of this article (10.1186/s12974-018-1135-3) contains supplementary material, which is available to authorized users.

Amyotrophic lateral sclerosis (ALS) is a systemic neurodegenerative disease characterized by a progressive loss of upper and lower motor neurons. Although the pathology of ALS is not yet sufficiently understood, there is considerable evidence that inflammatory processes play a major role in the pathogenesis of this disease [1–5].

Neuroinflammation in ALS is accompanied by infiltration of leukocytes from the peripheral blood [6]. However, it is still not clear whether these cells exhibit a destructive or a protective role during disease progression [2]. In a mouse model of ALS, elevated levels of the CC-chemokine ligand 2 (CCL2) and T cell infiltration were reported in the sciatic nerves in the slowly progressing ALS type (C57SOD1^G93A^ mice). On the contrary, this phenomenon was not observed in the fast-progressing 129SvSOD1G93A mice [7] suggesting a protective role of T cells during the disease course of ALS in this model.

In this study, we sought to gain insight into dysregulated inflammatory profiles of primary leukocytes derived from peripheral blood of patients with ALS (peripheral blood mononuclear cells, PBMC). To screen for alterations in bona fide molecules of immunological and inflammatory processes, we measured the expression of very late antigen-4 (VLA4), Toll-like receptor 4 (TLR4), CXC-chemokine receptor 3 (CXCR3), CC-chemokine receptor 5 (CCR5), CXC-chemokine receptor 4 (CXCR4), interferon-gamma (INF-γ), CC-chemokine receptor 2 (CCR2), and cluster of differentiation 11B (CD11B) [8, 9]. The surface expression of these molecules was detected on B cells, T cells, NK cells, and monocytes of 10 ALS patients and 10 age-matched healthy controls using multicolor flow cytometry (Fig. 1a). Clinical parameters of ALS patients are listed in the Additional file 1. We observed a significant upregulation of the chemokine receptors CXCR4 (*P* = 0.039), CXCR3 (*P* = 0.003), CCR2 (*P* = 0.006), and CCR5 (*P* = 0.002) on T cells from ALS patients compared to age-matched healthy controls (Fig. 1b). We also observed significant downregulation of the myeloid maturation marker CD11B (*P* = 0.023) and CCR2 (*P* = 0.045) the receptor for the major monocyte chemoattractant CCL2, in monocytes from ALS patients (Fig. 1c), affirming that dysregulations in CCR2 and CD11B may contribute to the pathophysiology of ALS via altered recruitment of microglia/monocytes and/or lymphocytes [4, 10–12]. A similar neuroprotective role of CCR2 by recruitment of microglia/monocytes has been shown in Alzheimer’s disease [13]. In our study, linear regression analysis revealed that the combination of the analyzed markers could significantly predict the categorization into ALS or healthy donors, with CXCR3 (*β* = − 0.50) and CCR5 (*β* = − 0.47) on T cells comprising the strongest predictors (adjusted *R*^2^ = 0.64, *F*(8,12) = 5.58, *P* = 0.004). Whether surface expression of these selected candidates is linked to disease progression and disease aggressiveness was ascertained by means of the D50 model of ALS progression, where D50 is derived from a sigmoidal curve fitted on all available ALSFRS-R values and represents the time in months to lose half of motor functionality (24 points of a maximum of 48 points) [14]. The surface expression of the chemokine receptor CXCR3 was significantly higher in slowly progressive ALS rather than in rapid progressive ALS cases (*U* test, *P* = 0.02). Further, there was a significant correlation between D50 and CXCR3 expression (Fig. 1d) promoting the hypothesis that a gain of function in T cells may have a protective function in ALS [1, 2, 12].

**Fig. 1:**
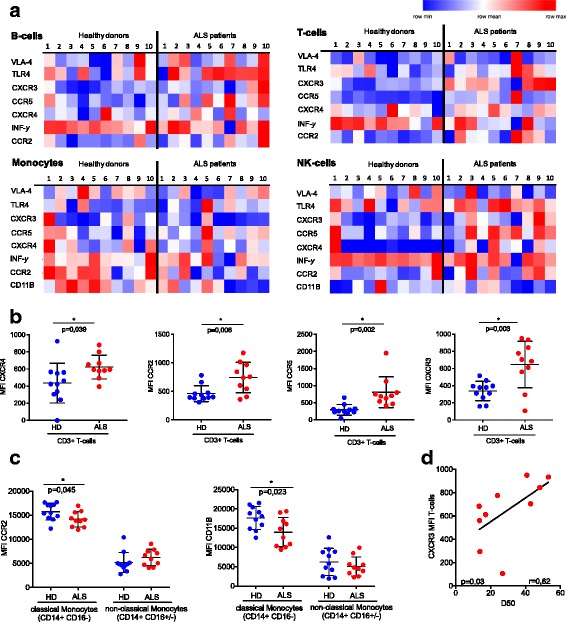
Alterations in chemokine receptor expression in peripheral blood samples. **a** Heat maps displaying the surface expression of selected inflammatory molecules measured by multicolor flow cytometry on ALS patient or healthy donor (HD)-derived B cells, T cells, monocytes, and NK cells, respectively. Low mean fluorescence intensity (MFI) values were indicated by blue color while high values are displayed in red. Heat maps were generated using the genepattern tool “HeatmapViewer” (Broad Institute, free ware tool). Statistics: unpaired *t* test. **b** Surface expression (MFI) of CXCR4, CCR2, CCR5, and CXCR3 on CD3+ T cells of ALS patients and healthy donors measured by flow cytometry. Statistical analysis was performed using unpaired *t* test (*n* = 10; *p < 0.05,). **c** MFI of CCR2 and CD11B on CD14 + CD16 classical monocytes and CD14+ CD16+ non classical monocytes of ALS patients and controls measured by flow cytometry (unpaired *t* test *n* = 10). **d** Aggressiveness of diseases calculated as D50 (period of time in months until the ALSFRS-R decreases to 24 points/50%)) correlated with the MFI of CXCR3 on the T cells from ALS patients measured by flow cytometry (spearman correlation, one-tailed test)

To further investigate if these changes at the expression level result in any changes in chemotactic behavior of lymphocytes, we conducted a chemotaxis assay using a modified Boyden chamber (experimental details are included in Additional file 1). PBMCs derived from ALS patients or healthy controls were placed in the upper chamber, while the respective chemoattractant (SDF1-α, IP-10, CCL2, RANTES or H_2_O control) was added to the lower well. All cells migrated to the lower well after 2.5 h were stained using lineage antibody and counted by flow cytometry. Although we observed a high number of migrating cells, using 100 ng/ml of the CXCR4 ligand SDF1-α as a chemoattractant, there was no significant difference in the numbers of migrated cells between healthy donors and ALS patients, nor could we find a relevant chemotactic response upon attraction with RANTES (*c* = 50 ng/ml) and CCL2 (*c* = 5 ng/ml) (Fig. 2a). In addition, chemoattraction using 50 ng/ml of the CXCR3-ligand IP-10 did not lead to a relevant migration of healthy donor cells, although PBMCs from some ALS patients showed a relevant chemotactic response for this stimulus, indicating a gain of function concerning IP-10-directed chemotaxis in these cells (Fig. 2a). In order to identify which cellular subset within the PBMC cell fraction is responsible for this observed effect, we analyzed for CD3+ T cells, CD56+ NK Cells, CD19+ B cells, and CD14+ monocytes using multicolor flow cytometry after chemoattraction with SDF1-α (Fig. 2b) and IP10 (Fig. 2c). Consistent with our observations using the total cell count of CD45+ cells, there were no major differences in the migration of healthy donor or ALS patient-derived cells upon chemoattraction with SDF-α among the different subsets analyzed (Fig. 2b). Following the use of IP10 as a chemoattractant, a trend for migration especially in the subset of T cells and to a smaller extent in NK cells and monocytes was seen in some ALS patients. Nevertheless, due to the limited number of patients analyzed, these effects did not reach statistical significance within the single subsets.

**Fig. 2:**
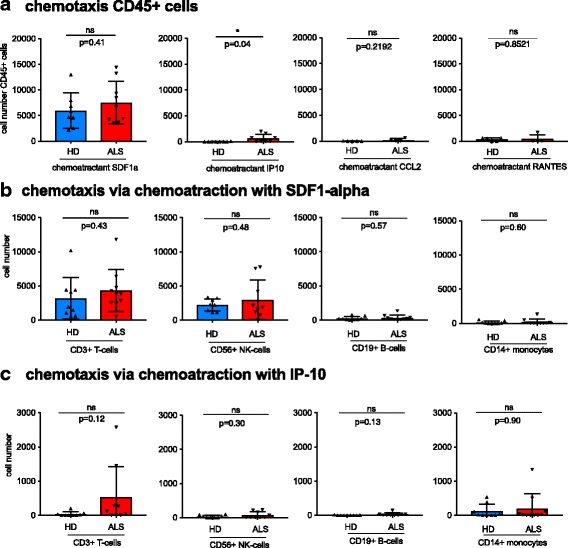
Migratory behavior of different cell types from ALS patients after chemoattraction with SDF1- α, CCL2, RANTES, and IP-10. **a** Chemotaxis displayed as total number of migrated cells (CD45+) in response to SDF1-α, IP-10, CCL2, or RANTES chemoattraction (calculated as cell count_chemokine_ − cell count_control_). Statistical analysis was performed using unpaired *t* test. **b** Chemotaxis of T cells, B cells, NK cells, and monocytes in response to chemoattraction with SDF1- α and **c** IP-10

Taken together, our data suggest a potential relevance of IP10-directed chemotaxis mediated by the CXCR3 receptor on peripheral blood lymphocytes in ALS. In order to estimate in how far the chemokine IP10 is may secreted in the CNS in ALS in vivo, we analyzed data from a publically available gene expression dataset in SOD1-G93A mice compared to age-matched wild-type mice [15]. Motoneurons derived from SOD1-G93A mice indeed show increased gene expression of IP10, whereas the expression of CXCR3 remains unchanged (Additional file 1: Figure S1). The potential therapeutic advantage of targeting chemokines and their receptors in ALS has been proven for SDF1-α-mediated chemotaxis, as the pharmacological blockade of CXCR4 significantly delayed disease progression in a mouse model [16]. According to the literature, CXCR3 has proinflammatory as well as immunosuppressive functions by altering regulatory T cells [17]. However, the role of CXCR3 or IP10 as therapeutic targets to influence disease progression in ALS needs to be verified in future studies using animal models of ALS.

## Additional file

Additional file 1: ALS patient information and supplemental material and methods. (DOCX 97 kb)

## Abbreviations

ALS: Amyotrophic lateral sclerosis
CCL2: CC-chemokine ligand 2
CCR2: CC-chemokine receptor 2
CCR5: CC-chemokine receptor 5
CD11B: Cluster of differentiation 11B
CXCR3: CXC-chemokine receptor 3
CXCR4: CXC-chemokine receptor 4
INF-γ: Interferon-gamma
IP10: Interferon gamma-induced protein 10 (CXCL10)
PBMC: Peripheral blood mononuclear cells
RANTES: Regulated on activation, normal T cell expressed and secreted (CXCL5)
SDF1-α: Stromal cell-derived factor 1 (CXCL12)
TLR4: Toll-like receptor 4
VLA4: Very late antigen-4
