# Supplementary material for: Dysregulation of chemokine receptor expression and function in leukocytes from ALS patients
Source: J Neuroinflammation. 2018 Mar 28;15:99. doi: 10.1186/s12974-018-1135-3 (PMC5874995; doi:10.1186/s12974-018-1135-3)
Supplement: Supplementary file 1 — ALS patient information and supplemental material and methods. (DOCX 97 kb) [file 12974_2018_1135_MOESM1_ESM.docx]

# **Supplemental Material:**

# ****

# **Supplemental Figure 1**: Gene expression of IP10 (left) and CXCR3 (right) in motoneurons of SOD1 G93A mice compared to wild type controls. The data was acquired using the Gene-expression-omnibus (GEO) platform. The data was generated by an Affymetrix mouse expression array (430A) and published in 2007[1].

# **participant information and methods:**

## **Subjects**

Peripheral blood samples were collected from 20 ALS patients and 24 age matched healthy donors. Exclusion criteria constituted the presence of any inflammatory conditions, including diabetes, multiple sclerosis, autoimmune diseases (e.g. psoriasis, ulcerative colitis), cancer and any current infections (as determined by C-reactive protein (CRP) and blood leucocyte counts). All blood samples were collected under informed consent according to the Declaration of Helsinki. Approximately 40ml of blood were obtained from each donor using EDTA for anticoagulation. Peripheral Blood Mononuclear Cells (PBMCs) were isolated using the Ficoll density gradient method (described in detail below) and subsequently used for flow cytometric and cell migration assays. Participant demographics and relevant clinical parameters are listed in detail in Tables 1 and 2. All ALS diagnoses were provided by attending physicians and in accordance with the El Escorial World Federation of Neurology criteria[2]. Based on collected Amyotrophic Lateral Sclerosis Functional Rating Scale-Revised (ALSFRS-R) scores, the following parameters were calculated for individual patients: progression rate (PR; calculated as 48 – ALSFRS-R score/disease duration), D50 (time taken in months for the individual’s ALSFRS-R score to drop to 24 points) and relative D50 (rD50; lone value describing individual disease covered in reference to D50, where 0 signifies disease onset and 0.5 indicates the time point of halved functionality). The D50 model and associated parameters have been previously established [3]. In brief, the D50 model makes it possible to capture and model the entire disease course for an individual in the context of his/her functional loss, thus allowing for unbiased sampling and comparisons between patients with vastly different disease courses.

| **Table 1** | **ALS patients** | **healthy controls** |
| --- | --- | --- |
| n | 20 | 24 |
| Age (years) mean ± SD | 59 ± 13 | 60 ± 13 |
| males | 12 | 7 |
| females | 8 | 15 |
| Mean ALSFRS-R ± SD | 74 ± 115 | / |
| Mean D50 ± SD | 39 ± 20 | / |
| Mean PR-FRS ± SD | 0,58 ± 0,51 | / |
| Mean rD50 ± SD | 0,42±0,17 | / |
| bulbar onset | 8 | / |
| limb onset | 10 | / |

## **Table 2: clinical information of ALS patients**

| **ALS patient** | **Experiment** | **gender** | **first manifestation** | **age at test (years)** | **ALSFRS-R** | **PR-FRS** | **D50** | **rD50** | **etiology** |
| --- | --- | --- | --- | --- | --- | --- | --- | --- | --- |
| 1 | expression | f | cervical | 81,4 | 35 | 0,81 | 23,9 | 0,33 | sporadic |
| 2 | expression | m | cervical | 53,9 | 48 | 0,00 | 13,6 | 0,11 | sporadic |
| 3 | expression | f | cervical | 53,4 | 21 | 1,80 | 13,4 | 0,56 | sporadic |
| 4 | expression | m | lumbosacral | 55,3 | 37 | 0,42 | 42,9 | 0,30 | sporadic |
| 5 | expression | m | bulbar | 65,8 | 32 | 1,07 | 17,1 | 0,44 | sporadic |
| 6 | expression | m | bulbar | 72,5 | 15 | 1,83 | 13,8 | 0,65 | sporadic |
| 7 | expression | m | bulbar | 77,0 | 32 | 0,67 | 26,9 | 0,45 | sporadic |
| 8 | expression | m | cervical | 38,9 | 44 | 0,16 | 48,2 | 0,26 | sporadic |
| 9 | expression | f | cervical | 39,9 | 14 | 0,39 | 53,1 | 0,82 | sporadic |
| 10 | expression | f | bulbar | 63,0 | 23 | 0,61 | 40,8 | 0,50 | sporadic |
| 11 | migration | f | lumbosacral | 66,3 | 23 | 0,69 | 35,4 | 0,51 | sporadic |
| 12 | migration | m | bulbar | 58,4 | 33 | 0,63 | 31,6 | 0,38 | sporadic |
| 13 | migration | f | bulbar | 66,3 | 44 | 0,25 | 49,9 | 0,16 | sporadic |
| 14 | migration | m | cervical | 58,4 | 32 | 0,23 | 72,6 | 0,48 | sporadic |
| 15 | migration | m | cervical | 41,3 | 30 | 0,31 | 68,9 | 0,43 | sporadic |
| 16 | migration | m | bulbar | 63,3 | 32 | 0,24 | 78,2 | 0,42 | sporadic |
| 17 | migration | f |  | 54,9 | 39 | 0,03 | 500,0 | 0,31 | autosomal-dominant juvenile ALS4 mutation in SEXT-gene |
| 18 | migration | f |  | 32,2 | 40 | 0,06 | 270,3 | 0,27 | juvenile ALS4 with heterozygote mutation in SEXT-gene |
| 19 | migration | m | cervical | 53,8 | 17 | 0,74 | 37,2 | 0,61 | sporadic |
| 20 | migration | m | bulbar | 78,0 | 30 | 0,72 | 32,5 | 0,38 | sporadic |

## **PBMC isolation using Ficoll-Paque density gradient centrifugation**

PBMCs were isolated from peripheral blood using Ficoll-Paque PLUS (GE Healthcare). EDTA blood was diluted with 1× PBS/2% FBS buffer and gently layered over 15ml of Ficoll-Paque PLUS . Gradients were centrifuged at 400 ×g for 25 min (brake turned off) at room temperature in a swinging rotor centrifuge. The mononuclear cell layer was carefully removed by pipetting and washed with 1xPBS/2%FBS by centrifugation at 250 x g for 10 min. PBMC pellets were suspended in Erylysis Buffer and incubated for 3 min on ice, diluted with 1xPBS/2% FBS and washed. Pellets were then suspended in 10ml of 1 × PBS/2% FBS and cells were counted using Trypan-Blue exclusion in a Neubauer chamber. PBMCs were then used for subsequent assays.

## **Flow cytometry**

Surface staining of PBMCs was conducted using fluorochrome-conjugated antibodies. For all flow cytometry experiments 3 million cells/tube were used from each donor; 5 tubes per donor were used in total. Lineage staining was performed with with anti-CD3 (PerCP-Cy5.5), CD14 (APC-Cy7), CD19 (Brill.Violett 650), CD56 (PE), and CD16 (Brill. Violett 650) in each tube. For analysis of chemokine receptors and integrins we used . anti-CXCR3 (AF488), CXCR4 (APC), CCR5 (Pe-Cy7), CCR2 (Pe-Cy7), CD11B (APC), VLA4 (Pe-Cy7) IFN-Gamma (AF488), TLR4 (PeCy7) as well as isotype controls for the respective colors. (Table 3) . Cells were incubated for 30 min under light protection. The Sytox blue dead cell stain was added to each tube shortly prior to analyses. Stained cells were measured with the BD FACSAria™ III cell sorter using the FACS DIVA (BD Biosciences) and FlowJo (LLC, USA) software packages.

**Table 3: antibodys used for Flow cytometry**

| **antibody** | **color** | **isotyp** | **manufacturer** | **clone** |
| --- | --- | --- | --- | --- |
| CD3 | PerCPCy5.5 | Mouse IgG, κ | Biolegend | Okt 03 |
| CD14 | APC-Cy7 | Mouse IgG, κ | Biolegend | HCD14 |
| CD19 | Brill-Violet 510 | Mouse IgG, κ | Biolegend | HIB19 |
| CD56 | PE | Mouse IgG, κ | Biolegend | HCD56 |
| CD16 | Brill. Violet 650 | Mouse IgG, κ | Biolegend | 3G8 |
| Isotyp control 1 | AF488 | Mouse IgG, k | Biolegend | MOPC-21 |
| isotyp control 2 | APC | Mouse IgG, κ | Biolegend | MOPC-21 |
| isotyp control 3 | PeCy7 | Mouse IgG, κ | Biolegend | MOPC-21 |
| Sytox blue dead cell stain | Pacific-blue | Kein Antikörper | Thermo Fischer | |
| CD11B | APC | Mouse IgG, κ | Biolegend | ICRF44 |
| VLA4 (CD49d) | PE-Cy7 | Mouse IgG, κ | Biolegend | 9F10 |
| CXCR3 | AF488 | Mouse IgG, κ | Biolegend | G025H7 |
| CXCR4 | APC | Mouse IgG, κ | Biolegend | 12G5 |
| CCR5 | PE-Cy7 | Mouse IgG, κ | Biolegend | J418F1 |
| IFN-gamma | AF488 | Mouse IgG | Novus Biologicals | 92101 |
| CCR2 | PeCy7 | Mouse IgG | Biolegend | K036C2 |
| TLR4 | PeCy7 | Mouse IgG, k | EBioscience | HTA125 |
| CD45 | APC | Mouse IgG, k | BD | HI30 |

## **Migration assay**

Post isolation, PBMCs were stimulated with 1 µg/ml Phytohaemaglutinin (PHA) and IL-2 50U/ml and incubated for 12 hours. Cells were counted and 400000 cells were added to each chamber in 100µl RPMI with 10%FBS. In the lower chamber we added 1 µl/ml H_2_O as control or 100 ng/ml SDF1α, 50 ng/ml CCL2, 5 ng/ml CCL5, or 50 ng/ml IP10. Cells were incubated for 2.5h at 37°C. Cells that had migrated to the lower compartment were counted using flow-cytometry. For the total cell counting and subset analysis we gated on CD45+ (APC), CD3+ cells (PerCP-Cy5.5), CD14+ (APC-Cy7), CD19+ (Brill.Violett 510) and CD56 + cells (PE).

## **Statistical analysis**

Microsoft Excel and the GraphPad Prism (V 7.0 GraphPad Software, USA) software package were used for statistical analyses. The unpaired t-test and Pearson’s test were used for assessing between-group comparisons and correlations, respectively. Statistical significance was set at p < 0.05.

1. Ferraiuolo, L., et al., *Microarray analysis of the cellular pathways involved in the adaptation to and progression of motor neuron injury in the SOD1 G93A mouse model of familial ALS.* J Neurosci, 2007. **27**(34): p. 9201-19.

2. Brooks, B.R., et al., *El Escorial revisited: revised criteria for the diagnosis of amyotrophic lateral sclerosis.* Amyotroph Lateral Scler Other Motor Neuron Disord, 2000. **1**(5): p. 293-9.

3. Poesen, K., et al., *Neurofilament markers for ALS correlate with extent of upper and lower motor neuron disease.* Neurology, 2017. **88**(24): p. 2302-2309.
